# Effects of standardized ileal digestible threonine to lysine ratio on growth performance of nursery, growing, and finishing pigs

**DOI:** 10.1093/tas/txag142

**Published:** 2026-09-17

**Authors:** Rafe Q Royall, Mike D Tokach, Jason C Woodworth, Joel M DeRouchey, Robert D Goodband, Jordan T Gebhardt, Carine M Vier, Matthew Spindler, Weasley A Orlando, Luis E Ochoa Zaragoza, Ning Lu, Wayne R Cast, Danielle F Wilson-Wells, Julia P Holen, Alyssa M Betlach

**Affiliations:** Department of Animal Sciences and Industry, College of Agriculture, Kansas State University, Manhattan, KS 66506-0201, United States; Department of Animal Sciences and Industry, College of Agriculture, Kansas State University, Manhattan, KS 66506-0201, United States; Department of Animal Sciences and Industry, College of Agriculture, Kansas State University, Manhattan, KS 66506-0201, United States; Department of Animal Sciences and Industry, College of Agriculture, Kansas State University, Manhattan, KS 66506-0201, United States; Department of Animal Sciences and Industry, College of Agriculture, Kansas State University, Manhattan, KS 66506-0201, United States; Department of Diagnostic Medicine/Pathobiology, College of Veterinary Medicine, Kansas State University, Manhattan, KS 66506-0201, United States; Genus PIC, Hendersonville, TN 37075, United States; Genus PIC, Hendersonville, TN 37075, United States; Genus PIC, Hendersonville, TN 37075, United States; Genus PIC, Hendersonville, TN 37075, United States; Genus PIC, Hendersonville, TN 37075, United States; Genus PIC, Hendersonville, TN 37075, United States; Genus PIC, Hendersonville, TN 37075, United States; SVC Research, St. Peter, MN 56082, United States; SVC Research, St. Peter, MN 56082, United States

**Keywords:** amino acid, grow-finish pig, lysine, threonine, requirement

## Abstract

The objective of these experiments was to evaluate the effect of standardized ileal digestible (SID) Thr:Lys ratio on growth performance of late nursery, growing, and finishing pigs. Four experiments were conducted using corn-soybean meal-based diets with pigs allotted to 5 or 6 treatments in randomized complete block designs. In Exp. 1, 987 late nursery pigs (initially 11.8 ± 0.32 kg) were used with 22 to 27 pigs per pen, 8 replications per treatment, and 5 SID Thr:Lys ratios from 53% to 68% of Lys. Increasing SID Thr:Lys ratio increased (linear, *P* ≤ 0.005) ADG and final BW. Average daily feed intake decreased then increased (quadratic, *P* < 0.001), therefore increasing then decreasing (quadratic, *P* < 0.001) G:F. In Exp. 2, 875 pigs (initially 43.4 ± 0.58 kg) were used with 21 to 27 pigs per pen, 7 replications per treatment, and 5 SID Thr:Lys ratios from 53% to 68% of Lys. Increasing SID Thr:Lys ratio increased (linear, *P* ≤ 0.037) ADG, final BW, and G:F. Dose-response models developed using the data from Exp. 1 and 2 predicted a requirement estimate for ADG beyond the highest dose tested (68% SID Thr:Lys ratio), which led us to test a wider range of SID Thr:Lys ratios in Exp. 3 and 4. In Exp. 3, 684 pigs (initially 37.4 ± 1.36 kg) were used with 9 or 10 pigs per pen, 12 replications per treatment, and 6 SID Thr:Lys ratios from 53% to 78% of Lys. Increasing SID Thr:Lys ratio increased then plateaued (quadratic, *P* ≤ 0.047) final BW, ADG, G:F and tended (quadratic, *P* = 0.093) to decrease then increase blood urea N (BUN). In Exp. 4, 662 pigs (initially 96.2 ± 1.04 kg) were used with 8 to 10 pigs per pen, 11 or 12 replications per treatment, and 6 SID Thr:Lys ratios from 53% to 78% of Lys. Increasing SID Thr:Lys ratio tended to increase then decrease (quadratic, *P* ≤ 0.063) final BW, ADG, and G:F, with the greatest numeric response observed between 63% and 68% SID Thr:Lys ratio. Data from Exp. 3 and 4 were combined and used to fit dose response curves for ADG, G:F, and BUN. A quadratic polynomial (QP) model suggested that ADG was maximized at 68.6% SID Thr:Lys, while a similar fitting broken-line linear (BLL) model suggested no further improvement in ADG beyond 61.1% SID Thr:Lys. A BLL model suggested no further improvement in G:F beyond 59.2% SID Thr:Lys. A QP model suggested that BUN was minimized at 69.2% SID Thr:Lys. These data suggest that SID Thr:Lys requirements are similar for pigs from 37 to 70 and 96 to 120 kg.

## Teaser

The results of these experiments suggest that a SID Thr:Lys ratio of 59% to 68% maximizes growth performance and feed efficiency in late nursery, growing, and finishing pigs.

## Introduction

Threonine is an essential amino acid (AA), which has been categorized as the first limiting AA for maintenance and development of intestinal tissue (Wang and Fuller 1989). This is primarily due to the substantial use of Thr by the portal drained viscera to produce proteins, such as mucins, that make up most of the mucous membrane, thus supporting gut integrity and immune function (Le Floc’h and Sève 2005; Schaart et al. 2005; Jayaraman et al. 2015). Additionally, the surface area and absolute volume of the gastrointestinal tract (GIT) increases dramatically with increasing BW (Elefson et al. 2021). As a result, mucin production should increase at a corresponding rate, thus suggesting an increase in the pigs’ Thr requirement to support both intestinal maintenance and growth performance. The NRC (2012) requirement estimate suggests that the dietary standardized ileal digestible (SID) Thr:Lys ratio should increase from 59% to 65% for pigs from 11 to 135 kg BW. However, subsequent research has observed a wide range of requirement estimates for SID Thr:Lys throughout the growing-finishing period. Studies conducted since 2012 in nursery and grower pigs have suggested SID Thr:Lys requirement estimates to maximize ADG and G:F ranging from 61% to 71%, with little correlation to increasing BW (Zhang et al. 2013 [22 to 50 kg]; Jayaraman et al. 2015 [7 to 18 kg]; Remus et al. 2019 [25 to 42 kg]). While there are a multitude of potential reasons for this variation, some include differences in environmental conditions, genetic potential for lean growth, and health status. Genetic improvement has resulted in increased protein accretion and growth performance; thus periodic reassessment of AA requirements is essential to allow pigs to reach their genetic potential. Therefore, the objective of these experiments was to evaluate the effect of varying SID Thr:Lys ratios on growth performance of late nursery, growing, and finishing pigs.

## Materials and methods

### Animal housing and management

The Kansas State University Institutional Animal Care and Use Committee approved the protocols used in these experiments (IACUC #4942 & #4525.2). Exp. 1 and 2 were conducted in a commercial research facility located in south-central Minnesota (SVC Research, St. Peter, MN). The barn had slatted concrete flooring, deep pits for manure storage, and was naturally ventilated. Pens (3 × 5.5 m) contained a 3-hole stainless steel dry self-feeder (Thorp Equipment, Thorp, WI) and a 1-cup waterer to provide ad libitum access to feed and water. In Exp. 1, the average external low temperature, high temperature, and relative humidity were 12°C, 22°C, and 50.8%, respectively. In Exp. 2, the average external low temperature, high temperature, and relative humidity were 19°C, 30°C, and 56.0%, respectively. Experiments 3 and 4 were conducted at the Kansas State University Swine Teaching and Research Center in Manhattan, KS. The facilities were totally enclosed and environmentally regulated. Each pen was equipped with a 2-hole dry, single-sided feeder (Farmweld, Teutopolis, IL) and a 1-cup waterer to provide ad libitum access to feed and water. Pens were located over a completely slatted concrete floor with a 1.21-m pit underneath for manure storage. In Exp. 3, the average external low temperature, high temperature, and relative humidity were 10°C, 21°C, and 43.5%, respectively. In Exp. 4, the average external low temperature, high temperature, and relative humidity were 19°C, 29°C, and 51.0%, respectively.

### Experimental diets

All treatment diets in Exp. 1 and 2 were manufactured at the United Farmers Cooperative Feed Mill (Klossner, MN). In Exp. 3 and 4, complete diets were manufactured at the O.H. Kruse Feed Technology Innovation Center (Manhattan, KS). In Exp. 1 and 2, dietary treatments contained SID Thr:Lys ratios of 53%, 58%, 62%, 65%, and 68%. Diets containing 53% and 68% SID Thr:Lys were blended to create the intermediate treatment diets (Table 1). Dietary SID Thr:Lys levels were selected such that they were below and above the PIC (2021) requirement estimates. In Exp. 3 and 4, dietary treatments contained SID Thr:Lys ratios of 53%, 58%, 63%, 68%, 73%, and 78%. The 53% and 78% SID Thr:Lys diets were blended to create the intermediate dietary treatments (Table 1). Dietary SID Thr:Lys levels were selected such that the estimated requirement was at the approximate midpoint of the titration based on the data from Exp. 1 and 2. The 73% and 78% treatments were added to ensure data points would be above the estimated requirement. For all experiments, daily feed additions to each pen were accomplished using a robotic feeding system (FeedPro, Feedlogic Corp., Wilmar, MN) able to record feed amounts for individual pens. All experimental diets were corn-soybean meal-based. Dietary nutrient composition and SID AA coefficients were derived from NRC (2012). Ratios of all essential AAs to Lys, except Thr, along with minerals were maintained at or above requirement estimates for the respective genetic line. The weight range of pigs, range of SID Thr:Lys ratios tested, genetic background of pigs, and location of each experiment are presented in Fig. 1.

**Figure 1:**
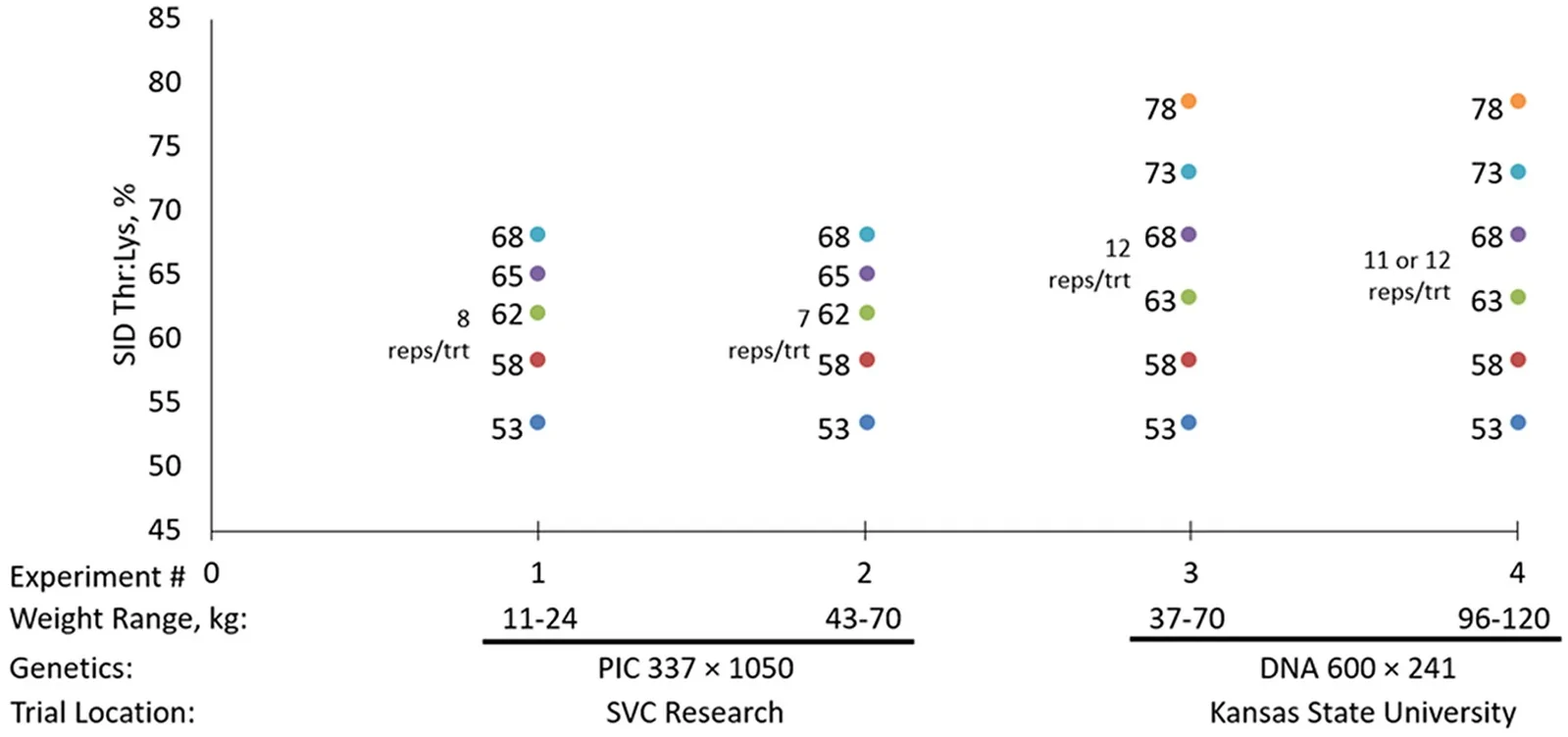
Summary of weight range, range of SID Thr:Lys ratios tested, genetic background of pigs, and location of each experiment.

**Table 1:** Diet composition (as-fed basis).

	Exp. 1**^a^**		Exp. 2**^b^**		Exp. 3**^c^**		Exp. 4**^d^**	
SID Thr:Lys, %	53	68	53	68	53	78	53	78
**Ingredient, %**								
** Corn**	64.86	64.67	78.39	78.17	79.56	79.33	90.08	89.77
** Soybean meal**	30.00	30.00	18.70	18.80	17.18	17.19	7.32	7.49
** Choice white grease**	1.45	1.45	—	—	—	—	—	—
** Monocalcium phosphate, 21% *P***	0.87	0.87	0.45	0.45	1.30	1.30	0.80	0.80
** Limestone**	1.00	1.02	1.18	1.18	0.75	0.75	0.83	0.83
** Sodium chloride**	0.61	0.61	0.55	0.55	0.50	0.50	0.50	0.50
** L-Lys-HCl**	0.36	0.36	0.28	0.28	0.31	0.31	0.28	0.28
** DL-Met**	0.23	0.23	0.11	0.11	0.07	0.07	—	—
** L-Thr**	0.03	0.20	—	0.12	—	0.22	—	0.14
** L-Trp**	0.02	0.02	0.01	0.01	0.03	0.03	0.03	0.03
** L-Val**	0.13	0.13	0.04	0.04	0.02	0.02	—	—
** Vitamin premix^f^**	0.25	0.25	0.25	0.25	0.15	0.15	0.10	0.10
** Trace mineral premix^g^**	0.15	0.15	0.15	0.15	0.15	0.15	0.08	0.08
** Copper sulfate**	0.06	0.06	—	—	—	—	—	—
**Total**	100	100	100	100	100	100	100	100
**Calculated analysis**								
** SID AA, %**								
** Lys, %**	1.15	1.15	0.81	0.81	0.84	0.84	0.58	0.58
** Ile:Lys**	56	56	59	59	61	61	60	60
** Leu:Lys**	119	119	131	131	144	144	170	170
** Met and Cys:Lys**	59	59	59	59	60	60	62	62
** Thr:Lys**	53	68	53	68	53	78	53	78
** Trp:Lys**	20	20	19.2	19.2	19.7	19.7	19.3	19.4
** Val:Lys**	70	70	70	70	71	71	73	73
** His:Lys**	36	36	37	37	42	42	45	46
** Arg: Lys**	102	102	106	106	97	97	91	92
** Phe and Tyr:Lys**	121	121	133	133	123	123	128	128
** Ala:Lys**	27	27	47	47	46	46	75	75
** Asp:Lys**	24	24	41	41	40	40	66	66
** Glu:Lys**	70	70	120	120	118	117	193	192
** Gly:Lys**	15	15	25	25	25	25	40	40
** Pro:Lys**	37	37	64	64	63	62	103	102
** Ser:Lys**	18	18	30	30	30	29	48	28
**NE, kcal/kg**	2,449	2,449	2,471	2,471	2,500	2,500	2,571	2,571
**SID Lys:NE, g/Mcal**	4.70	4.70	3.28	3.28	3.36	3.36	2.26	2.26
**CP, %**	18.7	18.8	14.2	14.3	15.1	15.3	11.2	11.4
**Nitrogen, %**	3.0	3.0	2.3	2.3	2.4	2.4	1.8	1.8
**Ca, %**	0.69	0.70	0.64	0.64	0.66	0.66	0.54	0.54
***P*, %**	0.57	0.57	0.43	0.43	0.61	0.61	0.46	0.46
**Avail. *P*, %**	0.39	0.39	0.27	0.27	0.34	0.34	0.22	0.22
**STTD *P*, %^h^**	0.44	0.44	0.33	0.33	0.44	0.44	0.26	0.26

a Diets were fed from 11.8 to 24.0 kg BW. The two diets were blended to create intermediate treatment diets containing 58, 62, and 65% SID Thr:Lys ratio.

b Diets were fed from 43.4 to 70.0 kg BW. The two diets were blended to create intermediate treatment diets containing 58, 62, and 65% SID Thr:Lys ratio.

c Diets were fed from 37.4 to 69.6 kg BW. The two diets were blended to create intermediate treatment diets containing 58, 63, 68, and 73% SID Thr:Lys ratio.

d Diets were fed from 96.2 to 120.4 kg BW. The two diets were blended to create intermediate treatment diets containing 58, 63, 68, and 73% SID Thr:Lys ratio.

e THR Pro; CJ America-Bio, Downers Grove, IL.

f Axtra PHY (Dupont, Wilmington, DE) provided 717 phytase units (FTU/kg) for an estimated release of 0.13% STTD P. Provided per kilogram of premix: 1,653,465 IU Vitamin A, 661,386 IU Vitamin D_3_, 17,637 IU Vitamin E, 1323 mg Vitamin K, 13 mg Vitamin B_12_, 19,842 mg niacin, 11,023 mg pantothenic acid, 3306 mg menadione.

g In Exp. 1, 2, and 3, the trace mineral premix provided: 110 mg/kg Zn, 110 mg/kg Fe, 33 mg/kg Mn, 16.5 mg/kg Cu, 0.3 mg/kg I, and 0.3 mg/kg Se. In Exp. 4 the trace mineral premix provided: 55 mg/kg Zn, 55 mg/kg Fe, 17 mg/kg Mn, 8 mg/kg Cu, 0.15 mg/kg I, and 0.15 mg/kg Se.

h Standardized total tract digestible.

### Experimental design

In each experiment, barrows and gilts were penned together with gender accounted for within pen. For all experiments, Thr was estimated to be the first-limiting AA, with Lys formulated to be approximately 10% below requirement estimates (PIC 2021) in Exp. 1 and 2 and approximately 10% below the requirement estimates for pigs raised in this facility (Royall et al. 2022) in Exp. 3 and 4. Between Exp. 1 and 2 and Exp. 3 and 4, all pens of pigs were fed a common diet for 23 and 28 d, respectively. Pens of pigs were then randomized to dietary treatment for the later experimental weight range.

### Experiment 1 (12 to 24 kg BW)

A total of 987 pigs (PIC 337 × 1050; initial BW 11.8 ± 0.32 kg) were used in a 21-d study with 22 to 27 pigs per pen with approximately equal numbers of barrows and gilts. Gates were adjusted such that each pig had approximately 0.61 m^2^ of pen space. Pens of pigs were blocked by BW and location and randomly assigned to 1 or 5 dietary treatments in a randomized complete block design (RCBD) with 8 replications per treatment. Following the conclusion of Exp. 1, all pigs were placed on a common diet for 23 days and then randomized to dietary treatment for Exp. 2.

### Experiment 2 (43 to 70 kg BW)

A total of 875 pigs (PIC 337 × 1050; initial BW 43.4 ± 0.58 kg) were used in a 28-d study with 21 to 27 pigs per pen with approximately equal numbers of barrows and gilts. Gates were adjusted such that each pig had approximately 0.61 m^2^ of pen space. Pens of pigs were blocked by BW and location and randomly assigned to 1 of 5 dietary treatments in a RCBD with 7 replications per treatment.

### Experiment 3 (37 to 70 kg)

Two groups of pigs were used in studies lasting 35 and 28 d, respectively, for a total of 684 pigs (DNA 600 × 241; initial BW 37.4 ± 1.36 kg). There were 9 or 10 pigs per pen with approximately equal numbers of barrows and gilts. Gates were adjusted such that each pig had approximately 0.74 m^2^ of pen space. Pens of pigs were blocked by BW and randomly allotted to 1 of 6 dietary treatments in a RCBD with 12 replications per treatment. Ten mL of blood was collected from the jugular vein of 3 randomly selected pigs per pen (2 barrows and 1 gilt) using a Monoject blood collection tube (Covidien, Minneapolis, MN). Blood was collected after a 12-h fasting period on d 14. Pigs were fasted for a 12-h period to minimize differences BUN caused by timing of last meal. Whole blood was centrifuged (1800 × g at 4°C for 30 min), and serum was collected and stored at -20°C until analysis. Serum was analyzed for urea N concentration using a Urea Nitrogen Colorimetric Detection Kit (Arbor Assays, Ann Arbor, MI). Following the conclusion of Exp. 3, all pigs were placed on a common diet for 23 days and then randomized to dietary treatment for Exp. 4.

### Experiment 4 (96 to 120 kg)

Two groups of pigs were used in 28 d studies, for a total of 662 pigs (DNA 600 × 241; initial BW 96.2 ± 1.04 kg). There were 8 to 10 pigs per pen with approximately equal numbers of barrows and gilts. Gates were adjusted such that each pig had approximately 0.74 m^2^ of pen space. Pens of pigs were blocked by BW and randomly allotted to 1 of 6 dietary treatments in a RCBD with 11 (58% SID Thr:Lys) or 12 replications per treatment. Similar blood collection and analysis procedures as in Exp. 3 were conducted, with the exception that blood was collected on d 13 and 14 in groups 1 and 2, respectively.

### Chemical analysis

The diets that were used for blending in Exp. 1 and 2 and all diets in Exp. 3 and 4 were analyzed for AA profile (Table 2). In all experiments, dietary samples were taken from each feeder approximately 3 d after the beginning of each trial. Diet samples were stored at −20°C until they were homogenized, subsampled and submitted for AA analysis (method 994.12; AOAC International 2012) conducted by Ajinomoto Animal Nutrition North America, Inc (Eddyville, IA) in Exp. 1, 2, and 3, and the University of Missouri-Columbia Agricultural Experiment Station Chemical Laboratories (Columbia, MO) in Exp. 4. Results of laboratory analysis indicated total nutrient profiles were consistent with expected formulated values (Table 2).

**Table 2:** Chemical analysis of diets (as-fed basis).^a^

	Exp. 1^b^		Exp. 2^b^		Exp. 3^b^						Exp. 4^c^					
SID Thr:Lys, %	53	68	53	68	53	58	63	68	73	78	53	58	63	68	73	78
**Crude protein, %**	19.6	17.9	15.0	15.7	15.1	15.0	15.7	15.4	15.1	15.4	10.6	10.5	10.6	10.8	11.0	11.1
**Essential amino acids, %**																
** Arg**	1.09	1.13	0.88	0.90	0.84	0.86	0.85	0.87	0.83	0.84	0.54	0.60	0.59	0.57	0.57	0.59
** His**	0.46	0.45	0.37	0.38	0.36	0.36	0.36	0.37	0.37	0.36	0.29	0.31	0.29	0.30	0.28	0.29
** Ile**	0.73	0.70	0.55	0.59	0.53	0.53	0.53	0.54	0.52	0.52	0.39	0.44	0.41	0.42	0.40	0.41
** Leu**	1.65	1.48	1.26	1.31	1.31	1.25	1.28	1.31	1.27	1.26	1.00	1.10	1.08	1.08	1.04	1.07
** Lys**	1.17	1.17	0.90	0.98	0.87	0.90	0.92	0.92	0.97	0.90	0.63	0.69	0.73	0.66	0.68	0.71
** Met**	0.40	0.48	0.30	0.41	0.30	0.30	0.30	0.30	0.29	0.29	0.19	0.21	0.22	0.19	0.19	0.20
** Met + Cys**	0.71	0.74	0.51	0.64	0.53	0.53	0.53	0.54	0.52	0.52	0.37	0.41	0.41	0.38	0.38	0.40
** Phe**	0.91	0.89	0.74	0.74	0.64	0.63	0.65	0.66	0.61	0.64	0.47	0.52	0.50	0.50	0.50	0.51
** Thr**	0.73	0.83	0.55	0.72	0.53	0.58	0.61	0.68	0.70	0.73	0.36	0.42	0.45	0.45	0.47	0.52
** Trp**	0.21	0.25	0.18	0.20	0.17	0.16	0.21	0.16	0.17	0.18	0.11	0.10	0.11	0.10	0.09	0.09
** Val**	0.87	0.88	0.65	0.74	2.16	2.11	2.12	2.09	2.11	2.11	0.48	0.53	0.53	0.51	0.51	0.53
**Non-essential amino acids, %**																
** Ala**	0.83	0.73	0.62	0.65	0.82	0.82	0.81	0.82	0.79	0.80	0.62	0.68	0.65	0.67	0.65	0.66
** Asp**	1.73	1.81	1.40	1.45	1.35	1.37	1.35	1.39	1.34	1.34	0.82	0.92	0.90	0.87	0.87	0.87
** Glu**	3.18	3.12	2.53	2.60	2.56	2.52	2.55	2.56	2.51	2.51	1.80	2.03	1.93	1.96	1.94	1.96
** Gly**	0.91	0.87	0.69	0.72	0.45	0.46	0.46	0.46	0.44	0.45	0.39	0.44	0.43	0.42	0.42	0.41
** Pro**	1.18	1.04	0.89	0.92	0.91	0.90	0.90	0.92	0.89	0.88	0.72	0.78	0.76	0.77	0.75	0.77
** Ser**	0.92	0.88	0.71	0.73	0.71	0.71	0.70	0.72	0.69	0.70	0.42	0.47	0.45	0.46	0.46	0.46
** Tyr**	0.44	0.42	0.30	0.32	0.21	0.20	0.22	0.23	0.20	0.22	0.34	0.38	0.36	0.36	0.35	0.38

a Diet samples were taken from all feeders approximately 3 d after the beginning of the trial and stored at −20°C. Values are reported on a total analyzed basis.

b Composite samples were submitted to Ajinomoto Heartland Inc. (Eddyville, IA) for amino acid analysis.

c Composite samples were submitted to University of Missouri-Columbia Agricultural Experiment Station Chemical Laboratories (Columbia, MO) for amino acid analysis.

### Statistical analysis

Data were analyzed as a randomized complete block design for a one-way ANOVA using the GLIMMIX procedure of SAS (v. 9.4, SAS Institute, Inc., Cary, NC) within each experiment. Pen was considered the experimental unit, pen average BW as a blocking factor, and treatment as the fixed effect in the statistical model. Linear and quadratic contrast coefficients were created with each experiment. Results were considered significant with *P* ≤ 0.05 and marginally significant with *P* ≤ 0.10. For Exp. 1 and 2, dose response curves were evaluated using quadratic polynomial (QP), broken-line linear (BLL), and broken-line quadratic (BLQ) models using the NLMIXED procedure within SAS. The best-fitting model was selected using the Bayesian Information Criterion (BIC) with improved model fits accepted when BIC decreased at least 2.0.

Data from Exp. 3 and 4 were combined and used to fit dose response curves for ADG, G:F, and BUN as a function of SID Thr:Lys ratio. Dose response curves were evaluated using the same methodology as in Exp. 1 and 2. In all dose-response models, the linear predictor included SID Thr:Lys ratio as an explanatory variable and block nested within experiment as a random effect. In addition, linear and quadratic terms for SID Thr:Lys ratio and their interaction with experiment were tested prior to fitting dose response models. There was no evidence of an interaction between SID Thr:Lys ratio and experiment and the interactive terms were eliminated from the models.

## Results

### Experiment 1 (11- to 24- kg PIC 337 × 1050 pigs)

Increasing SID Thr:Lys ratio increased (linear, *P* < 0.005) ADG and final BW (Table 3). Average daily feed intake decreased then increased (quadratic, *P* < 0.001) and G:F increased then decreased (quadratic, *P* < 0.001) as SID Thr:Lys ratio increased. A QP model predicted that ADG was maximized at levels greater than the highest SID Thr:Lys ratio tested (68% SID Thr:Lys), while a QP model suggested that G:F was maximized at 61.8% SID Thr:Lys (Supplemental Figs. 1 and 2, available as supplementary data at *Translational Animal Science* online).

**Table 3:** Effects of increasing SID Thr:Lys ratio on growth performance of 11- to 24-kg pigs, Exp. 1.^a^

	SID Thr: Lys ratio, %						*P* =	
Item:	53	58	62	65	68	SEM	Linear	Quadratic
**BW, kg**								
** d 0**	12.0	11.7	11.8	11.6	11.9	0.32	0.554	0.111
** d 21**	23.1	23.7	24.1	24.0	24.5	0.41	0.005	0.766
**Growth performance**								
** ADG, g**	527	547	563	569	585	10.8	<0.001	0.948
** ADFI, g**	847	780	782	787	870	21.2	0.599	<0.001
** G:F, g/kg**	622	706	726	729	673	23.1	0.007	<0.001

a A total of 987 pigs (PIC 337 × 1050, initially 11.8 ± 0.32 kg) were used in a 21-d growth performance study with 22 to 27 pigs per pen and 8 replications per treatment.

### Experiment 2 (43- to 70-kg PIC 337 × 1050 pigs)

Increasing SID Thr:Lys increased (linear, *P* ≤ 0.037) ADG, final BW, and G:F (Table 4). Quadratic polynomial models suggested that both ADG and G:F ratio were maximized at greater than 68% SID Thr:Lys. Similarly fitting BLL models predicted no further improvement in ADG and G:F beyond 67% and 65% SID Thr:Lys, respectively (Supplemental Figs. 3 and 4, available as supplementary data at *Translational Animal Science* online).

**Table 4:** Effects of increasing SID Thr:Lys ratio on growth performance of 43- to 70-kg pigs, Exp. 2.^a^

	SID Thr:Lys ratio, %						*P* =	
Item:	53	58	62	65	68	SEM	Linear	Quadratic
**BW, kg**								
** d 0**	43.3	43.6	43.3	43.3	43.3	0.58	0.841	0.958
** d 28**	68.5	68.6	69.0	69.8	70.0	0.80	0.037	0.510
**Growth performance**								
** ADG, g**	900	898	914	947	942	16.6	0.020	0.524
** ADFI, kg**	2.09	2.05	2.10	2.10	2.13	0.031	0.214	0.325
** G:F, g/kg**	431	438	435	450	443	5.1	0.027	0.841

a A total of 875 pigs (PIC 337 × 1050 initially 43.4 ± 0.58 kg) were used in a 28-d growth performance study with 21 to 27 pigs per pen and 7 replication per treatment.

### Experiment 3 (37- to 70-kg DNA 600 × 241 pigs)

Increasing SID Thr:Lys ratio increased (quadratic, *P* ≤ 0.025) ADG and final BW up to 68% SID Thr:Lys ratio with no improvement thereafter (Table 5): however, ADFI was not affected (*P* > 0.10). As a result, increasing SID Thr:Lys ratio improved (quadratic, *P* = 0.047) G:F, with the greatest improvement seen when increasing from 53% to 58% SID Thr:Lys ratio with incremental improvements thereafter. Blood urea N tended (quadratic, *P* = 0.093) to decrease then increase with increasing SID Thr:Lys ratio.

**Table 5:** Effects of increasing SID Thr:Lys ratio on growth performance of 37- to 70-kg pigs, Exp. 3.^a^

	SID Thr:Lys ratio, %							*P* =	
Item:	53	58	63	68	73	78	SEM	Linear	Quadratic
**BW, kg**									
** Initial**	37.4	37.4	37.4	37.4	37.4	37.4	1.36	0.990	0.884
** Final**	67.7	68.9	69.4	69.6	69.2	69.2	0.88	0.030	0.025
**Growth performance**									
** ADG, g**	960	994	1,017	1,022	1,008	1,009	12.3	0.004	0.005
** ADFI, kg**	2.22	2.22	2.24	2.24	2.23	2.18	0.036	0.530	0.256
** G:F, g/kg**	432	450	454	457	453	463	4.1	<0.001	0.047
**Blood urea nitrogen, mg/dL^b^**	7.26	7.17	7.13	6.88	7.08	7.14	0.205	0.215	0.093

a Two groups of pigs were used in studies lasting 35 and 28 d, respectively, for a total of 684 pigs (DNA 600 × 241; initially 37.4 ± 1.36) with 9 or 10 pigs per pen and 12 replications per treatment.

b Blood samples were taken on d 14 from 3 pigs per pen (2 barrows and 1 gilt).

### Experiment 4 (96- to 120-kg DNA 600 × 241 pigs)

Final BW tended to increase (quadratic, *P* = 0.061) with increasing SID Thr:Lys ratio. Average daily gain increased (linear, *P* = 0.035 and quadratic, *P* = 0.063) with increasing SID Thr:Lys ratio with the greatest ADG at 68% SID Thr:Lys ratio (Table 6). Increasing SID Thr:Lys ratio tended to increase (quadratic, *P* = 0.061) G:F, with the greatest improvement when increasing from 53% to 58% SID Thr:Lys ratio and incremental improvements thereafter. Additionally, increasing SID Thr:Lys ratio linearly decreased (*P* = 0.016) and tended to quadratically decrease (*P* = 0.059) BUN with increasing SID Thr:Lys ratio with the lowest BUN at 68% SID Thr:Lys ratio.

**Table 6:** Effects of increasing SID Thr:Lys ratio on growth performance of 96- to 120-kg pigs, Exp. 4.^a^

	SID Thr:Lys ratio, %							*P* =	
Item:	53	58	63	68	73	78	SEM	Linear	Quadratic
**BW, kg**									
** Initial**	96.2	96.3	96.2	96.2	96.2	96.2	1.04	0.962	0.985
** Final**	118.8	119.5	120.0	120.4	119.8	119.8	1.07	0.136	0.061
**Growth performance**									
** ADG, g**	801	831	850	863	839	850	19.7	0.035	0.063
** ADFI, kg**	2.82	2.82	2.84	2.89	2.91	2.85	0.038	0.114	0.301
** G:F, g/kg**	284	295	299	298	288	298	5.2	0.106	0.061
**Blood urea nitrogen, mg/dL^b^**	6.67	6.52	6.39	6.34	6.42	6.40	0.150	0.016	0.059

a Two groups of pigs were used in 28 d studies, for a total of 662 pigs (DNA 600 × 241; initially 96.2 ± 1.04 kg) with 8 to 10 pigs per pen and 11 or 12 replications per treatment.

b Blood samples were taken on d 13 and 14, group 1 and 2, respectively, from 3 pigs per pen (2 barrows and 1 gilt).

### Combined dose response curves Exp. 3 and 4

The dose-response models for Exp. 1 and 2 predicted a requirement estimate for ADG beyond the highest dose tested (68% SID Thr:Lys ratio). Thus, the greater range of SID Thr:Lys ratios was used in Exp. 3 and 4. The data from Exp. 3 and 4 was combined to develop dose response models to estimate the SID Thr:Lys requirement for 37- to 120-kg DNA 600 × 241 pigs. A QP model suggested that ADG was maximized at 68.6% SID Thr:Lys, while a similarly fitting BLL model suggested no further improvement in ADG beyond 61.1% SID Thr:Lys (Fig. 2). A BLL model suggested no further improvement in G:F beyond 59.2% SID Thr:Lys (Fig. 3). For BUN, a QP model suggested that BUN was minimized at 69.2% SID Thr:Lys (Fig. 4).

**Figure 2:**
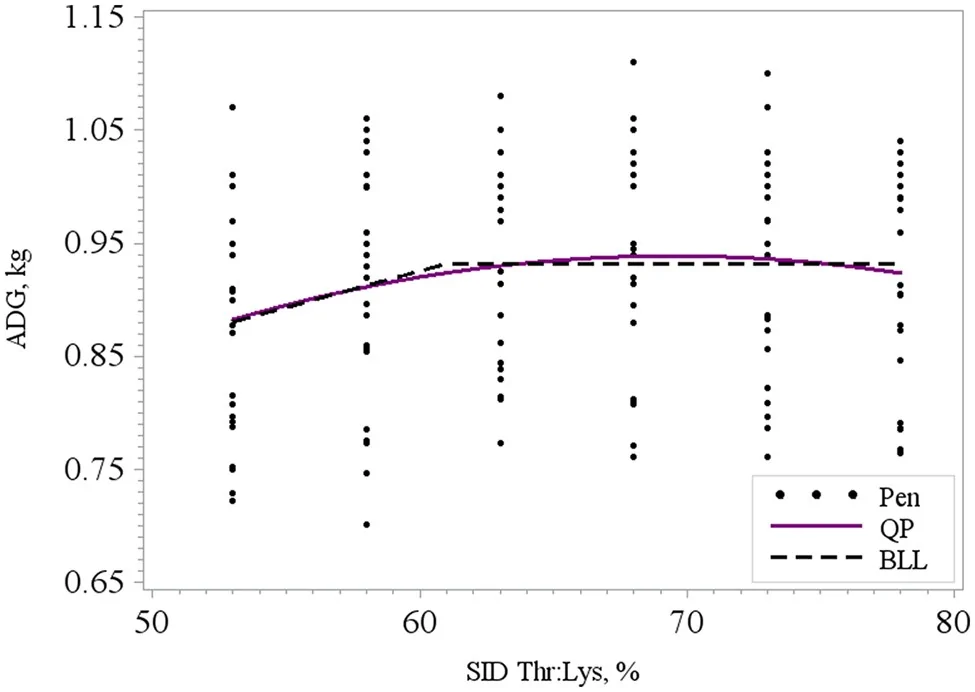
Estimation of SID Thr:Lys ratio requirements to maximize ADG for 37- to 120-kg DNA 600 × 241 pigs, Exp. 3 and 4. The QP and BLL models resulted in the best fit, based BIC (BIC = -386.2 vs. -386.7, QP vs. BLL). the QP model predicted 95 and 100% of maximum ADG at 53.7 and 68. SID Thr:Lys, respectively. The developed QP model equation for ADG was: ADG = −0.00021 × (SID Thr:Lys, %)^2^ + 0.02881 × (SID Thr:Lys, %) – 0.06193. The BLL model predicted no further improvement beyond 61.1% SID Thr:Lys.

**Figure 3:**
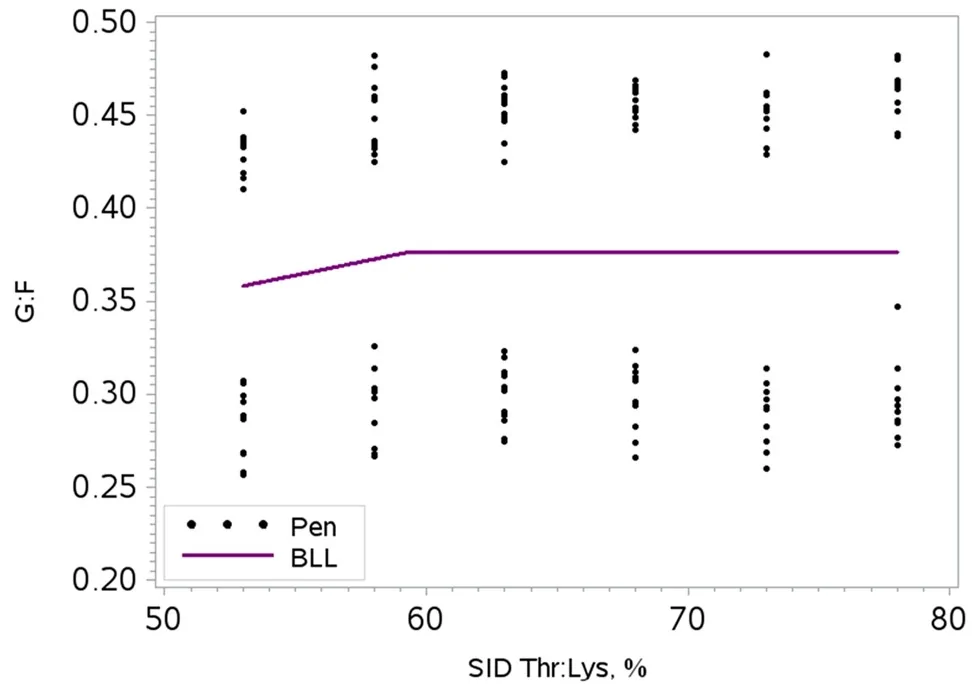
Estimation of SID Thr:Lys ratio requirements to maximize G:F for 37- to 120-kg DNA 600 × 241 pigs, Exp. 3 and 4. The BLL model resulted in the best fit, based on BIC. The BLL model predicted no further improvement beyond 59.2% SID Thr:Lys.

**Figure 4:**
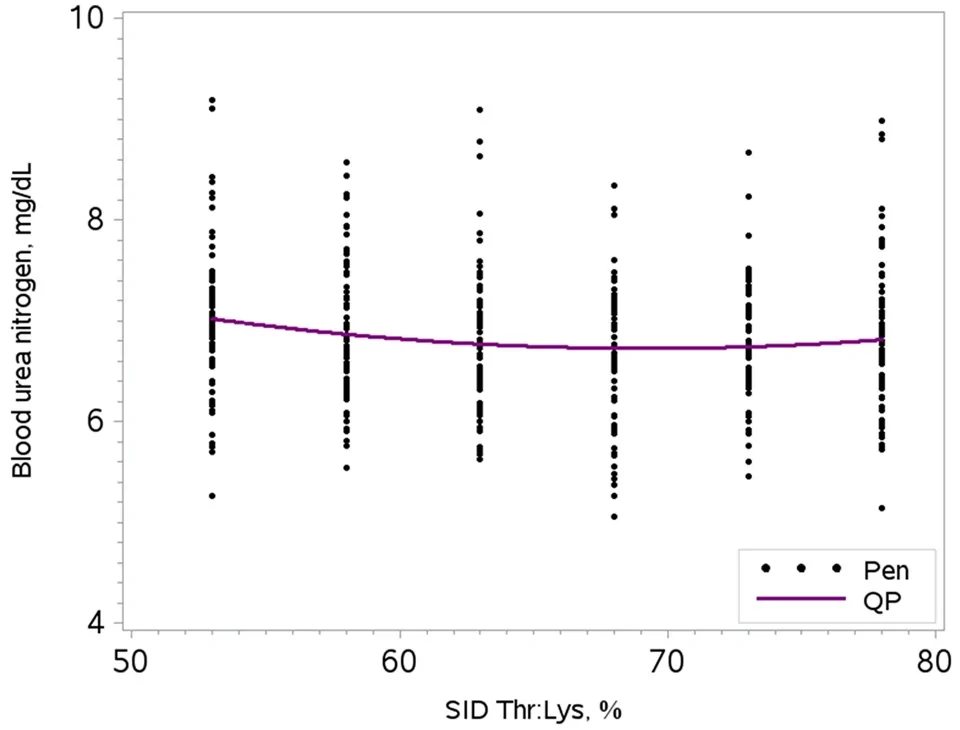
Estimation of SID Thr:Lys ratio requirements to minimize BUN for 37- to 120-kg DNA 600 × 241 pigs, Exp. 3 and 4. The QP model resulted in the best fit, based on BIC. The QP model predicted 95 and 100% of minimum BUN at 51.9 and 69.2% SID Thr:Lys, respectively. The developed QP model equation for BUN was: BUN mg/dL = 0.001121 × (SID Thr:Lys, %)^2—^0.1552 × (SID Thr:Lys, %) + 12.0988.

## Discussion

Threonine has been reported as the second limiting AA in corn-soybean meal-based diets and has been determined to be the first limiting AA for maintenance in swine (Wang and Fuller 1989). Schaart et al. (2005) observed that 40% to 60% of dietary Thr intake is extracted by the portal drained viscera during first pass metabolism, with the majority being used by epithelial tissue for production of mucins, accounting for approximately 71% of total Thr usage, while the remaining 29% is either used for muscle protein synthesis or oxidized (Le Floc’h and Sève 2005; Tang et al. 2021). As Thr is preferentially utilized within the GIT for mucin production, its deficiency results in restricted skeletal muscle growth (Munasinghe et al. 2017).

In two studies utilizing pigs inoculated with either *Escherichia coli* or *Salmonella typhimurium*, Wellington et al. (2018, 2019b) observed that SID Thr:Lys ratios between 68% and 72% of Lys supported maximum ADG, G:F, and protein deposition during an enteric infection. These results reinforce the necessity of Thr for protein synthesis and maintenance in the intestinal tissue. Thus, as intestinal capacity, or intestinal protein turnover, increases as the pig grows, mucin production should theoretically increase as well, which has led to the hypothesis that the pigs’ Thr requirement for maintenance and growth should increase with increased BW. The NRC (2012) estimates suggest that SID Thr:Lys requirements increase from 59% to 65% as BW increases from 11 to 135 kg.

Multiple studies conducted in the 1990s observed requirement estimates for total Thr:Lys ratio under 60% in 9- to 23-kg pigs (Adeola et al. 1994; Bergstrom et al. 1996; Ragland and Adeola 1996). Research conducted more recently throughout the growing-finishing period has found more varied responses. In 22- to 50-kg pigs, Zhang et al. (2013) observed that ADG and G:F were maximized at 70% and 68% SID Thr:Lys, respectively, while Mathai et al. (2016) suggested that ADG and G:F were maximized at 66% and 63% SID Thr:Lys in pigs of a similar weight range. However, experiments conducted by Jayaraman et al. (2015), Remus et al. (2019), and Wellington et al. (2018, 2019a, 2019b) observed requirement estimates ranging from 61% to 72% SID Thr:Lys to maximize ADG, G:F, and protein deposition. This variation in requirement estimates led us to further study the response to various SID Thr:Lys levels.

Similar to previous studies, we observed a wide range of requirement estimates across our first two experiments. In Exp. 1 (11 to 24 kg) we observed that ADG and G:F were maximized at >68% and 61.8% SID Thr:Lys, respectively, based on QP models. These requirement estimates are considerably higher than results in much of the published research conducted in a similar weight range (Adeola et al. 1994; Bergstrom et al. 1996; Jayaraman et al. 2015). For early finishing pigs (43 to 70 kg) in Exp. 2, we observed that ADG and G:F were maximized at or above 67% and 65%, respectively. However, in each of our first two experiments, the highest SID Thr:Lys ratios tested (68% of Lys) may not have been high enough to obtain a clear quadratic response as the QP models suggest the maximum response would be observed beyond our tested levels. As a result, we conducted Exp. 3 and 4, in which we tested a wider range of SID Thr:Lys ratios to determine requirement estimates from 37- to 120-kg pigs. Data from Exp. 3 and 4 were analyzed to determine whether the requirement for SID Thr:Lys increases with increasing BW as suggested by the NRC (2012). The absence of linear or quadratic interactions between SID Thr:Lys ratio and experiment suggest that the SID Thr:Lys requirement remains consistent for 37- to 120-kg pigs. The dose response models suggest that ADG is maximized at 61.1% to 68.6% SID Thr:Lys and G:F is maximized at 59.2% SID Thr:Lys ratio. These results are consistent with previous research that has estimated the SID Thr:Lys requirement between 63% and 71% (Zhang et al. 2013; Mathai et al. 2016; Remus et al. 2019).

The wide range in predicted requirement estimates for a response variable is likely due to the different models selected. Broken-line models are typically more conservative, or suggest a lower requirement estimate than QP models. This is because broken-line models specify that there are only small-to-no improvements in a response variable when increasing beyond a certain nutrient level. Meanwhile QP models frequently suggest a greater requirement estimate than a broken-line model, as a QP model considers incremental increases in a response variable as a nutrient level increases. Each model has its role in determining a requirement estimate; however, because of the different assumptions in response to an increasing nutrient, it confirms that there is no one set requirement estimate.

Pigs have a limited ability to store free AA, and as such excess AA are catabolized to urea or used as an energy source (Rosell and Zimmerman 1985; Chang et al. 2000). Thus, many studies have used BUN as a measure to estimate an AA requirement, as BUN concentrations increase in response to a deficiency, or excess, of one or more AA (Li et al. 1998; Wang et al. 2007; Zhang et al. 2013). Therefore, utilizing models to determine the SID Thr:Lys concentration required to minimize BUN can help determine the point at which pigs most efficiently synthesize protein. In Exp. 3 and 4, a QP model suggested that BUN was minimized at 69.2% SID Thr:Lys, regardless of weight range. These results are almost identical to the 68.6% SID Thr:Lys ratio that maximized ADG and are higher than 62% SID Thr:Lys required to minimize BUN in 22- to 50-kg pigs observed by Zhang et al. (2013). These differences could be a result of genetic differences, increased BW in our studies, or greater protein accretion potential in the pigs used in our studies.

In conclusion, these results would suggest a range of SID Thr:Lys ratio requirement estimates depending on response criteria. In 37- to 120-kg pigs, our dose response models suggest that ADG is maximized at 61.1% to 68.6% SID Thr:Lys, G:F is maximized at 59.2% SID Thr:Lys ratio, and BUN is minimized at 69.2% SID Thr:Lys ratio. However, our observations that SID Thr:Lys ratio and BW did not have a linear or quadratic interaction (Exp. 3 and 4) suggest that SID Thr:Lys requirements to maximize growth performance and minimize BUN may not increase with increasing BW.

## Supplementary Material

txag142_Supplementary_Data

## List of abbreviations

AA: amino acid
ADFI: average daily feed intake
ADG: average daily gain
BIC: Bayesian Information Criterion
BLL: broken-line linear
BLQ: broken-line quadratic
BUN: blood urea nitrogen
BW: body weight
CP: crude protein
DM: dry matter
G:F: gain-to-feed
QP: quadratic polynomial
RCBD: randomized complete block design
SID: standardized ileal digestibility
STTD: standard total tract digestibility
